# Exploratory study on microRNA profiles from plasma-derived extracellular vesicles in Alzheimer’s disease and dementia with Lewy bodies

**DOI:** 10.1186/s40035-019-0169-5

**Published:** 2019-10-04

**Authors:** Ana Gámez-Valero, Jaume Campdelacreu, Dolores Vilas, Lourdes Ispierto, Ramón Reñé, Ramiro Álvarez, M. Pilar Armengol, Francesc E. Borràs, Katrin Beyer

**Affiliations:** 1 Department of Pathology, Health Sciences Research Institute Germans Trias i Pujol (IGTP), Universitat Autònoma de Barcelona, Badalona, 08916 Spain; 2 REMAR-IVECAT group, Health Sciences Research Institute Germans Trias i Pujol (IGTP), 08916 Badalona, Spain; 3 Department of Neurology, Hospital Universitari de Bellvitge, L’Hospitalet de Llobregat, 08907 Spain; 4 Department of Neurology, Hospital Universitari Germans Trias i Pujol, 08916 Badalona, Spain; 5 Genomic and Microscopy facilities, Health Sciences Research Institute Germans Trias i Pujol (IGTP), 08916 Badalona, Spain; 6 Department of Cell Biology, Physiology and Immunology, Universitat Autònoma de Barcelona (UAB), Barcelona, Spain

**Keywords:** Neurodegenerative disorders, Biomarker, Exosomes, Next generation sequencing, Extracellular vesicles

## Abstract

**Background:**

Because of the increasing life expectancy in our society, aging-related neurodegenerative disorders are one of the main issues in global health. Most of these diseases are characterized by the deposition of misfolded proteins and a progressive cognitive decline. Among these diseases, Alzheimer’s disease (AD) and dementia with Lewy bodies (DLB) are the most common types of degenerative dementia. Although both show specific features, an important neuropathological and clinical overlap between them hampers their correct diagnosis. In this work, we identified molecular biomarkers aiming to improve the misdiagnosis between both diseases.

**Methods:**

Plasma extracellular vesicles (EVs) -from DLB, AD and healthy controls- were isolated using size-exclusion chromatography (SEC) and characterized by flow cytometry, Nanoparticle Tracking Analysis (NTA) and cryo-electron microscopy. Next Generation Sequencing (NGS) and related bibliographic search was performed and a selected group of EV-associated microRNAs (miRNAs) was analysed by qPCR.

**Results:**

Results uncovered two miRNAs (hsa-miR-451a and hsa-miR-21-5p) significantly down-regulated in AD samples respect to DLB patients, and a set of four miRNAs (hsa-miR-23a-3p, hsa-miR-126-3p, hsa-let-7i-5p, and hsa-miR-151a-3p) significantly decreased in AD respect to controls. The two miRNAs showing decreased expression in AD in comparison to DLB provided area under the curve (AUC) values of 0.9 in ROC curve analysis, thus suggesting their possible use as biomarkers to discriminate between both diseases. Target gene analysis of these miRNAs using prediction online tools showed accumulation of phosphorylation enzymes, presence of proteasome-related proteins and genes involved in cell death among others.

**Conclusion:**

Our data suggest that plasma-EV associated miRNAs may reflect a differential profile for a given dementia-related disorder which, once validated in larger cohorts of patients, could help to improve the differential diagnosis of DLB versus AD.

**Electronic supplementary material:**

The online version of this article (10.1186/s40035-019-0169-5) contains supplementary material, which is available to authorized users.

## Background

Neurodegenerative disorders with largest economic burden in our society include dementia syndromes such as Alzheimer’s disease (AD), frontotemporal dementia, dementia with Lewy bodies (DLB), and movement disorders namely Parkinson’s disease (PD), in which about 50% of patients may develop dementia 15 years after PD onset [1–3]. Clinically, along the course of the three diseases, a progressive cognitive decline affecting the normal and social functions of the patients is observed [4]. Moreover, neuropathologically, a common neurodegeneration-related feature is the deposition of misfolded proteins. While in DLB and PD α-synuclein accumulations, named Lewy bodies are found [5], in AD –and also in about half of all DLB cases- ß-amyloid senile plaques and hyperphosphorylated tau are accumulated in the brain [6, 7]. Correspondingly, DLB shows an important neuropathological and therefore clinical overlap with both, PD and AD, hampering its correct identification. Currently, a high proportion of DLB cases are missed or misdiagnosed as AD, resulting in an incorrect treatment of the patient, which leads to the development of adverse reactions in 50% of these treated patients [7].

Over the past decade, the role of extracellular vesicles (EVs) in the development and functioning of the central nervous system has been deeply explored [8–10]. EVs, such as exosomes (EXs) and microvesicles (MVs), seem to be important effectors in the development of cognitive and neurodegenerative disorders. They have been shown to mediate the transport of prions and misfolded pro-aggregating proteins from cell to cell [11–14]. Because EVs are produced by each individual cell and provide a protected environment to shuttle not only proteins but also microRNAs (miRNAs) to the intercellular space and to different body fluids, the study of their content has emerged as an area of interest in the biomarker field, also in neurodegenerative disorders [15–18]. To date, several EV-associated miRNAs have been identified as altered and related to neurodegenerative disorders [19–21]. Many of these studied EVs were obtained from cerebrospinal fluid (CSF) samples. However, due to several issues on CSF-collection (difficulty, invasiveness, morbidity, risk), alternative sources such as plasma-derived EVs have been lately reported [22, 23].

In this study, we analysed the miRNA profile associated to plasma-derived EVs from DLB, AD and healthy controls. We further investigated whether differences in the plasma-EV-miRNA content could be of help to better characterize neurodegenerative disorders, specifically DLB and AD. Our data suggest that some plasma-EV associated miRNAs are differentially found in DLB and AD patients, and thus could help to improve the differential diagnosis of these overlapping neurodegenerative disorders.

## Materials and methods

### Blood collection and sample processing

The Clinical Research Ethics Committee of our institution approved the following protocol and from each subject, written informed consent was obtained according to the Declaration of Helsinki Principles [24]. DLB patients (*n* = 18; age range 62–84 years; mean 72.5 years; male: female ratio 1:1.75) were recruited by neurologists specialized in Lewy body disorders at the Dementia Unit, Department of Neurology from the University Hospital Bellvitge, (L’Hospitalet de Llobregat, Barcelona). Diagnosis of DLB patients was established according to the 2005 DLB Consortium Criteria [25], and the age at onset was defined as the age when parkinsonism or memory loss was first reported by patients’ relatives. A group of age- and gender-matched healthy individuals (*n* = 15; age-range 61–85; mean 70.5 years; male: female 1:2) from the same hospital were also recruited. Finally, a group of AD patients (*n* = 10; age range 62–80; mean 71; male: female ratio 1:1.5) was enrolled by the Neurology Department of the University Hospital Germans Trias i Pujol. AD diagnosis was assessed following the 2011 revised criteria from the National Institute on Aging and the Alzheimer’s Association [26]. These patients presented a Global Deterioration Scale of 4.3 ± 1.2 degrees. Clinical data of all patients and healthy controls enrolled in this study are shown in Table 1.

**Table 1:** Data of patients and control individuals included in the study

Sample	Clinical Diagnosis	DatScan	Gender	Age (blood coll)^a^	Age at onset	MMSE^b^	APOE
Ex1	DLB	abnormal	F	85	83	20	33
Ex2	DLB	abnormal	M	79	73	24	23
Ex3	DLB	positive	F	82	78	15	34
Ex4	DLB	positive	F	73	68	16	33
Ex5	DLB	no^c^	F	90	84	5	34
Ex6	DLB	positive	F	80	64	10	33
Ex7	DLB	positive	F	86	79	28	33
Ex8	DLB	no	F	79	74	19	34
Ex9	DLB	positive	M	74	67	6	33
Ex10	DLB	positive	F	79	74	12	33
Ex11	DLB	positive	M	65	59	5	33
Ex12	DLB	positive	M	77	67	24	33
Ex13	DLB	positive	F	83	80	16	33
Ex14	DLB	positive	M	70	62	12	33
Ex15	DLB	positive	F	77	73	18	34
Ex16	DLB	positive	M	63	59	15	33
Ex17	DLB	positive	F	64	62	11	na^d^
Ex18	DLB	normal	M	73	73	22	34
Ex19	AD	–	F	75	74	18	34
Ex20	AD	–	M	75	75	22	23
Ex21	AD	–	F	70	70	23	33
Ex22	AD	–	F	80	80	20	33
Ex23	AD	–	F	70	63	12	33
Ex24	AD	–	M	62	60	16	34
Ex25	AD	–	M	72	65	15	34
Ex26	AD	–	F	74	70	22	34
Ex27	AD	–	M	64	na	18	34
Ex28	AD	–	F	68	na	20	34
C-Ex1	CTRL	–	F	71	–	28	33
C-Ex2	CTRL	–	F	67	–	29	33
C-Ex3	CTRL	–	F	66	–	27	33
C-Ex4	CTRL	–	F	75	–	28	34
C-Ex5	CTRL	–	F	74	–	26	33
C-Ex6	CTRL	–	M	69	–	30	33
C-Ex7	CTRL	–	M	72	–	27	23
C-Ex8	CTRL	–	F	69	–	28	33
C-Ex9	CTRL	–	F	67	–	26	34
C-Ex10	CTRL	–	M	67	–	28	23
C-Ex11	CTRL	–	F	72	–	27	33
C-Ex12	CTRL	–	F	69	–	29	33
C-Ex13	CTRL	–	F	61	–	28	33
C-Ex14	CTRL	–	M	73	–	27	33
C-Ex15	CTRL	–	M	85	–	26	33

^a^age at blood collection; ^b^MMSE: The Mini-Mental State Examination; ^c^no DaTSCAN evaluation available; ^d^not available

Blood samples from all participants were obtained following ISEV- International Society for Extracellular Vesicles- guidelines [27] and applying the same collection protocol in both hospitals. In short, 15 mL of peripheral blood were collected by venous puncture using a 21-gauge needle coupled to a butterfly device and using sodium citrate pre-treated tubes (BD Vacutainer, New Jersey, USA) as described previously [28]. After discarding the first 2–3 ml, blood was collected and mixed with the anticoagulant by gently inverting tubes gently. Samples were processed within the first 2 h after collection. Plasma was clarified of platelets and cells by consecutive centrifugation steps at 500 x g for 10 min, 2500 x g for 15 min and a last step at 13,000 x g for 10 min. Plasma samples were frozen in a freezing container with freezing rate of - 1 °C/min and kept at − 80 °C until EV purification. Samples did not suffer from more than 2 freeze-thaw cycles.

### EV isolation by size exclusion chromatography (SEC)

Size Exclusion Chromatography (SEC) was used for isolating plasma-EVs as previously described [28]. Briefly, Sepharose-CL2B (Sigma Aldrich, St Louis, MO, USA) was stacked in a Puriflash column Dry Load Empty 12 g (20/pk) from Interchim (France)-Cromlab, S.L. (Barcelona, Spain). Once the column was completely stacked, two mL of plasma (previously thawed on ice) were loaded onto the column and eluted with filtered PBS. A total of 20 fractions (0.5 mL each) were immediately collected.

### EV characterization

EV fractions were characterized for their protein concentration, presence of specific EV-markers, size and morphology.

As published before, protein concentration for each SEC collected fraction was measured by absorbance at 280 nm in a Thermo Scientific Nanodrop® ND-100 (Thermo Fisher Scientific, Waltham, MA). Also, SEC-fractions were analysed for the presence of CD9, CD81, CD63 and CD5L as specific EV-markers by bead-based flow cytometry assay [28, 29]. Briefly, 50 μL of each fraction were incubated with 0.5 μL aldehyde/sulphate-latex beads-4 μm (Invitrogen, Carlsbad, CA) for 15 min at room temperature, re-suspended in coupling buffer and incubated overnight. After two washing steps with the same buffer, EV-coated beads were incubated with anti-CD9 (Clone VJ1/20), anti-CD63 (Clone TEA 3/18), anti-CD5L (ab45408 from Abcam) and anti-CD81 (clone 5A6, from Santa Cruz Biotech) or polyclonal IgG isotype (Abcam, Cambridge, UK) for 30 min at 4 °C. After a washing step, samples were subjected to 30 min incubation at 4 °C with a FITC-conjugated secondary goat anti-mouse antibody (Southern Biotech, Birmingham, AL) for CD9, CD81 and CD63, and secondary Ab anti-rabbit AlexaFLuor 488 (Invitrogen, Carlsbad, CA) for CD5L. Two final washing steps were performed before analysing the samples by flow cytometry in a FacsVerse cytometer (BD Biosciences, New Jersey, USA). Mean fluorescence intensity (MFI) values were plotted (Flow Jo software, Tree Star, Ashland, OR) and tetraspanin-positive fractions with the highest MFI (fractions 10–12 from our SEC column) were considered as EV-containing fractions and pooled for the forthcoming analysis.

Aiming to check EV morphology and size, EV-enriched pools were also subjected to cryo-electron microscopy (cryo-EM) and to Nanoparticle Tracking Analysis (NTA) (*n* = 6), as reported earlier [28].

### Isolation of microRNA

A volume of 750 μL of pooled EVs from each sample was lyophilized at − 23 °C /overnight and used for miRNA extraction using the miRCURY^TM^ RNA Isolation Kit-biofluids (Exiqon Vedbaek, Denmark) at room temperature as described by the manufacturer. Briefly, lyses solution and protein precipitation solution were added to each sample. After incubation at room temperature for 1 min, samples were centrifuged at 11,000 x g for 3 min. Isopropanol was added to the supernatants and the mix was transferred to the provided columns. After a first centrifugation at 11,000 x g for 30 s, several washing steps at the same centrifugation speed with 1BF and 2BF solutions were applied. The column was subjected to a last 2 min centrifugation to completely dry the membrane. MiRNA elution was performed by adding 100 μL of RNase-free H2O directly onto the membrane and centrifuging it for 1 min at 11,000 x g. The obtained material was kept at − 80 °C until further analysis.

In samples utilized for RT-qPCR validation experiments, 2 artificial RNAs (UniSp4 and UniSp5 from the RNA spike-in kit, Exiqon, Vedbaek, Denmark) were spiked into the lysis buffer before miRNA purification, according to the manufacturer’s protocol enabling the assessment of miRNA purification and amplification efficiency.

### MicroRNA discovery by next generation sequencing (NGS) and raw data analysis

The total volume of the obtained miRNAs from 7 DLB and 7 control samples was precipitated overnight at − 20 °C with 1 μL of glycogen (20 μg/ μL), 10% 3 M AcNa (ph 4.8) and 2 volumes of absolute ETOH. miRNAs were re-suspended in RNase free H2O and heated at 65 °C for 2–3 min. Quality control and size distribution of the purified small RNA was assessed by Bioanalyzer 2100, (Agilent Technologies, Santa Clara, USA).

The whole precipitated volume of each sample (10 μL) was used for library preparation by NEBNext Multiplex Small RNA Sample Preparation Set for Illumina (New England Biolabs, Massachusetts, USA) following kit instructions. Individual libraries were subjected to the quality analysis using a D1000 ScreenTape (TapeStation, Agilent Technologies), quantified by fluorimetry and pooled. Clustering and sequencing were done in an Illumina Sequencer (MiSeq, Illumina, San Diego, USA) at 1 x 50c single read mode and 200,000 reads were obtained for each sample. Obtained FastQ raw data were analysed as follow: (1) Trimmomatic was used to remove the adapter sequences from the reads [30]; (2) Reads were mapped to the human genome using Bowtie2 algorithm and individual miRNAs were identified [31]; (3) For each sample, the number of reads mapped to a particular miRNA sequence was counted; and (4) the total count of reads was normalized applying the weighted trimmed means of M-values (TMM) [32]. Before differential expression analysis, Lilliefors’ composite goodness-of-fit test, Jarque-Bera hypothesis test and Shapiro-Wilk test were applied to check the normality of our samples.

For NGS expression analysis the following criteria were followed: at least minimum of 5 reads per sample when present to considered a miRNA as present; present in all patient samples (minimum of 5 reads) and absent (less than 5 reads) in more than half of the control cohort; present in all control samples and absent in more than half of the patients for presence/absence consideration; present in most samples from both cohorts but differentially expressed between both groups. Differential expression analysis for DLB versus control cohorts was performed by Wilconson-rank sum test (*p*-value < 0.05) [33] and validated by the Leave-One-Out (LOO) cross-validation methodology.

### Bibliographic search and miRNA selection

Literature search based on PubMed and The Nervous System Disease NcRNAome Atlas (NSDNA) [34] databases was performed aiming to identify miRNAs already described as deregulated in dementia and neurodegenerative disorders. We then combined this bibliographic information with our data from NGS results in plasma-EV from DLB and healthy control samples. Thus, a group of 15 highly represented miRNAs (all of them producing more than 5000 reads in the NGS assay and belonging to the top most abundant miRNAs identified by NGS) were further considered for expression studies to compare AD and DLB by qPCR.

### Reverse transcription and qPCR analysis

MiRCURY LNA^TM^ Universal cDNA synthesis Kit II and miRNA PCR system Pick & Mix (Exiqon, Vedbaek, Denmark) were used for cDNA synthesis and qPCR validation analysis of the selected miRNAs according to the manufacturer’s instructions (see Additional file 1: Table S1 for further purchasing information). Due to the low concentration of genetic material associated to vesicles, 2 μL of undiluted miRNAs were used for retrotranscription at 42 °C for 60 min. A third spike-in miRNA (UniSp6) from the same kit (Exiqon) was used as retrotranscription control. After enzyme inhibition at 95 °C for 5 min, cDNA was diluted 1:80 and 4 μL were used for qPCR reaction with ExiLENT SYBR Green Master Mix (Exiqon, Vedbaek, Denmark) on a LightCycler 480 (Roche, Basel, Switzerland), following kit’s instructions. For each sample, miRNAs were analysed in duplicate and the mean value was used in next data analyses. Amplification of the used spike-ins (UniSp4, UniSp5, and UniSp6) was performed in the same PCR pre-designed panels (Exiqon, Vedbaek, Denmark) with an interplate calibrator control miRNA (UniSp3).

### Data analysis and statistical testing

All statistical analyses were performed using Prism 7 (GraphPad Software, Inc., CA, USA).

Regarding EV characterization, MFI values for EV-markers, EV-concentrations, and EV-size are given as mean ± SD. Two-tailed unpaired T-test was applied (*p* < 0.05 was considered as statistically significant).

For qPCR analysis, Cq (quantification cycle) values were determined for each qPCR and the average of duplicates was obtained. The variability between the different plates was corrected by the Cq values for UniSp3 as interplate calibrator. As stable genes, RNA-isolation control spike-in UniSp4 and the retro-transcription control spike-in UniSp6 were considered as reference genes. The average of the two Cq values was used as reference value to calculate miRNA expression. Relative expression in DLB and AD was estimated compared to healthy controls and represented as fold expression changes obtained by 2^-ΔΔCt^. Considering the low number of samples, multiple comparisons between the three groups (DLB, AD and controls) were performed using the Kruskal-Wallis non-parametric test and Dunn’s test was used for multiple corrections. A *p*-value below 0.05 was considered statistically significant. One-variable area under the ROC curve (AUC) was calculated considering the expression change in order to assess the possible diagnostic potential of each differentially expressed miRNA by the Wilson/Brown method (GraphPad Prism v7; 95% C.I., AUC > 0.750 was assessed as minimum value to be considered as a good-potential biomarker).

We have submitted all relevant data of our experiments to the EV-TRACK knowledgebase (EV-TRACK ID: EV180020) [35].

### microRNA target prediction

A list of possible affected target genes for the differentially expressed miRNAs by qPCR was obtained by miRGate [36] database. Filtering of the software output data was performed following the instructions of the developers. We first considered validated targets, named “confirmed predictions” and showing the number of confirmed databases of gene-miRNA biding supporting this prediction group in the output data. For the remaining suggested targets, we took into account the column named “computational predictions”, defined by the number of computational methods supporting this prediction group. These referred to genes bioinformatically predicted to be regulated by the given miRNA, although no validation has been reported. Target with more than one computational prediction were also considered [36].

The relationship between the miRGate validated targets (those predicted with more than 1 confirmed predictions) were analysed with String [37] and Panther Gene Ontology [38] Databases, obtaining an integrated clustered network based on biological processes, cellular components and KEEG (Kyoto Encyclopaedia of genes and genomes) Pathways. In both cases, default settings were used. In Panther GO-analysis, Fisher’s exact test was applied and Bonferroni correction for multiple testing was used.

## Results

### EV isolation and characterization

**Fig. 1:**
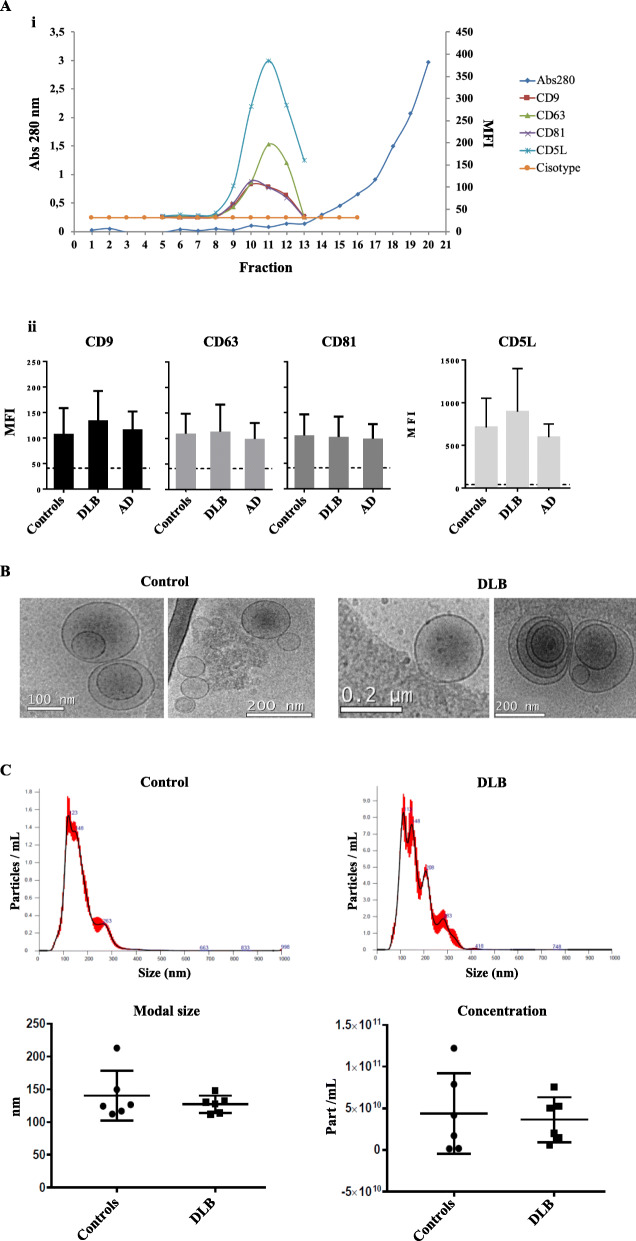
Isolation and characterization of plasma-EVs. **a** EVs are eluted in low protein SEC fractions and EV-markers could be detected by bead-based flow cytometry. An example graph for one of the processed samples is shown (**i**); MFI values of the tetraspanins CD9, CD63 and CD81, and marker CD5L in the SEC-pooled fractions are represented. Bars represent mean +/−SD of 15 independent experiments in the case of tetraspanins and 5 in the case of CD5L (**ii**); **(b)** Cryo-EM images with characteristic shape and size of isolated vesicles; **(c)** above: representative NTA profiles for one control-EV and one DLB-EV sample, below: concentration and modal distribution for 6 control and 6 DLB samples (mean ± SD)

EVs were isolated from 2 mL of platelet-depleted plasma by SEC from three different cohorts (healthy controls, DLB and AD), obtaining 20 fractions of 0.5 mL from each sample. EV-fractions were identified by the presence of tetraspanins CD9, CD81, and CD63. Alternatively, some samples were also profiled for the presence of CD5L, recently described as plasma-EV marker [39]. In all cases, EV-enriched fractions –positive marked for the 4 EV markers used- were eluted, preceding the bulk of soluble protein (Fig. 1A i). Three fractions showing the highest MFI values of each sample were pooled for further experiments. No differences in MFI and expression of EV-markers were found between the three groups (Fig. 1A ii). Some samples were submitted to cryo-electron microscopy analysis confirming the presence of vesicles with the expected size and morphology (Fig. 1b). NTA data did not show any difference in particle concentration or particle size (Fig. 1c) between DLB and control groups.

### Discovery phase: miRNA profiles of DLB and control plasma-EVs

**Fig. 2:**
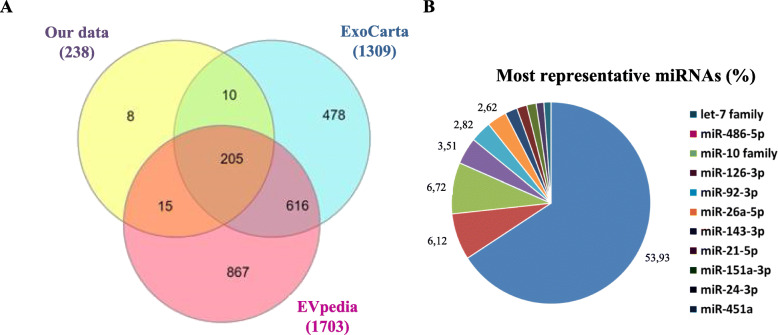
Profiling and characterization of NGS data. **a** Mature miRNAs identified were compared to the already described as human-EV content in ExoCarta and EVpedia; **b** Hsa-let-7 family was the most representative miRNA family in our data set

As first approach (discovery phase), 7 samples of DLB patients and 7 samples of age-matched controls were included for library construction and NGS analysis. An average of 4,364,157 ± 647,775 raw reads per sample was obtained for control samples (Additional file 2: Table S2); for DLB, we obtained 4,307,581 ± 2,076,192 reads per sample. Although 311 mature miRNAs were identified among all samples, only those showing at least 5 reads (*n* = 238) were further considered. More than 95% of these miRNAs were already included in vesicular databases EVpedia [40] and ExoCarta [41] as related to EV or exosomes from human samples (Fig. 2a). The hsa-let-7 family appeared as one of the most representative miRNA groups among our data set (Fig. 2b). The whole list of all identified miRNA has been submitted to the EVpedia database and entitled as this manuscript.

Statistical analyses of the 238 miRNAs identified by NGS showed no differences between DLB and controls (Wilcoxon *p*-value > 0.05 for all).

### Selection of dementia related miRNAs for further qPCR analysis

Despite the lack of differences and statistical significance between DLB and healthy control cohorts, we carried out a bibliographic search for those miRNAs that yielded more than 5000 reads in the NGS experiment. Their association to dementia, DLB, AD and PD was examined and a group of 15 miRNAs (Table 2), mainly associated to dementia and AD, was further analysed by qPCR in three independent cohorts of samples.

**Table 2:** Selected microRNAs for further qPCR validation analysis

miRNA	Previously reported in the literature	Ref	NGS Counts	Mean Fold change (2^-^^ΔΔCt^) (95% C.I.)		
				CTRL	DLB	AD
hsa-miR-21-5p	Down-regulated in serum-EVs from AD patients compared to controls	[20]	14,337	1.49 (0.91–2.06)	1.81 (0.67–2.96)	0.29 (0.03–0.56)
	Up-regulated in serum-EVs from PD patients in comparison to AD					
	Down-regulated in CSF from AD patients compared to control individuals	[42]				
	Down-regulated in plasma from PD patients compared to normal controls	[43]				
hsa-miR-26a-5p	Deregulated in AD blood (different results in NGS and qPCR)	[44]	27,896	1.88 (0.96–2.79)	1.04 (0.54–1.53)	0.8 (0.09–1.5)
	miR-26a is up-regulated in CSF from PD patients compared to controls	[21]				
	Up-regulated in blood from AD patients compared to controls	[45, 46]				
	Down-regulated in CSF from AD patients compared to controls	[19]				
	Down-regulated in serum from AD patients compared to healthy controls	[47]				
	Down-regulated in ALS blood compared to controls	[48]				
hsa-let-7i-5p	Down-regulated in PD brains compared to controls	[49]	15,170	1.43 (0.78–2.09)	1.23 (0.05–1.75)	0.41 (0.06–0.75)
	Increased expression in AD patients’ hippocampus	[50]				
	Up-regulated in CSF from AD compared to controls.	[42]				
	Down-regulated in ALS compared to controls	[48]				
hsa-miR-126-3p	miR-126 is down-regulated in CSF-EXs from AD and PD patients vs controls	[21, 51]	37,418	2.23 (1.41–3.04)	1.88 (0.66–3.1)	0.89 (0.03–1.74)
	Increased expression in the hippocampus of AD mouse model vs WT controls	[52]				
hsa-miR-451a	Up-regulated in serum-EXs from MS patients compared to controls	[53]	10,058	2.05 (1.21–2.88)	1.84 (0.76–2.92)	0.19 (0.05–0.33)
	Increased in plasma from vascular dementia patients compared to healthy controls	[54]				
	Decreased expression in CSF-EXs from AD compared to controls	[55]				
	Down-regulated in ALS compared to controls	[48]				
hsa-miR-23a-3p	Up-regulated in brain tissue from AD patients	[50]	6834	1.85 (1.24–2.45)	1.17 (0.65–1.68)	0.52 (0.06–0.97)
	miR-23a is down-regulated in serum samples from AD patients’ vs FTD and controls	[56]				
	Down-regulated in blood from MS patients compared to controls	[57]				
	Down-regulated in CSF from AD patients compared to control individuals	[42]				
	Reflect MS disease status in serum-EXs	[53]				
	Increased expression in brain tissue from AD patients compared to controls	[51]				
	Down-regulated in ALS blood compared to controls	[48]				
hsa-let-7f-5p	Up-regulated in AD hippocampus compared to healthy controls	[50]	191,299	1.29 (0.59–1.99)	1.28 (0.23–2.34)	1.05 (0.33–1.76)
	Up-regulated in AD serum compared to healthy controls	[58]				
	Down-regulated in blood from AD patients compared to controls	[45]				
	Down-regulated in ALS blood /plasma compared to controls	[48, 59]				
hsa-miR-409-3p	Down-regulated in the prefrontal cortex of AD patients	[50]	5236	1.48 (0.05–3.01)	1.46 (0.12–2.79)	1.35 (0.19–2.52)
	Down-regulated in CSF from PD patients compared to controls	[20]				
	Up-regulated in CSF-EXs from PD patients compared to AD and control EXs	[21, 60]				
	Up-regulated in serum-EXs from MS patients compared to controls	[53]				
	Down-regulated in plasma from vascular dementia patients compared to controls	[54]				
hsa-miR-92a-3p	Down-regulated expression in serum from PD patients compared to controls	[61]	30,066	1.89 (0.42–3.35)	1.22 (0.54–1.89)	1.38 (− 0.29–3.06)
	Differentially expressed in PD and Huntington patients’ brain	[62]				
	Up-regulated in CSF from AD patients compared to control individuals	[42]				
	Down-regulated in the serum samples of AD patients’ vs MCI subjects	[63]				
	Differentially expressed in AD and MCI	[64]				
hsa-let-7b-5p	Let-7b miRNA is up-regulated in AD patients’ brain	[51]	107,394	1.98 (0.07–3.89)	0.81 (0.39–1.23)	2.097 (0.58–3.61)
	Let-7b is down-regulated in the white matter of AD patients	[65]				
	Increased amounts of let-7b in CSF from AD patients	[66]				
	Differentially expressed in AD in comparison to controls	[64]				
hsa-miR-151a-3p	Up-regulated in AD blood compared to controls	[44]	5798	2.04 (− 0.13–4.22)	0.98 (0.34–1.63)	0.70 (− 0.09–1.5)
	Up-regulated in blood from AD patients compared to controls	[45]				
	Differentially expressed in AD in comparison to controls	[64]				
hsa-miR-24-3p	miR-24 is up-regulated in serum and plasma of MSA compared to PD patient**s**	[67]	10,896	1.5 (0.68–2.33)	1.49 (0.28–2.69)	25.21 (3.11–47.31)
	Dow-regulated in plasma-EXs from AD patients compared to controls	[22]				
	miR-24 is deregulated in CSF from AD patients	[68]				
	Decreased expression in AD-CSF compared to controls	[42, 69]				
	miR-24 expression is decreased in CSF from PD patients compared to controls	[16]				
	Differently expressed in blood and CSF in AD and FTD patients	[70]				
hsa-miR-143-3p	miR-143 is up-regulated in AD brain patients	[51]	17,380	5.85 (− 2.88–14.6)	1.22 (− 0.11–2.56)	2.42 (− 1.28–6.11)
	Down-regulated in CSF from ALS patients compared to controls	[71]				
	Increased expression in serum-EXs of AD patients vs healthy controls	[23]				
	Down-regulated in serum from AD patients	[72]				
	Up-regulated in CSF from AD and dementia patients compared to controls	[42]				
	Up-regulated in brain of PD mouse model	[73]				
	Increased expression in serum ALS compared to controls	[74]				
hsa-miR-423-5p	Under-represented in the cortex of AD patients	[50, 65]	8928	1.99 (0.52–3.46)	1.23 (0.43–2.04)	8.04 (2.02–14.06)
	Increased expression in CSF from AD and dementia patients vs controls	[42]				
	Down-regulated expression in PD putamen tissue	[73]				
	Differentially expressed in AD blood compared to healthy controls	[64]				
	Down-regulated in plasma from PD patients compared to normal controls	[43]				
	Up-regulated in CSF from AD patients compared to controls	[51]				
	Low expression in CSF from PD patients	[75]				
hsa-miR-183-5p	In serum, associated to neurofibrillary tangles score in AD patients	[20]	3820	1.18 (0.63–1.73)	0.79 (0.15–1.44)	15.77 (0.84–30.7)
	Differentially expressed in AD and other types of dementia patients vs controls	[42]				
	Down-regulated in peripheral blood from ALS patients	[48]				
	Decreased expression is associated to PD	[76]				

Literature search was performed in different databases for the top most abundant miRNAs found by the exploratory Next Generation Sequencing (NGS) study. PubMed, and the Nervous System Disease NcRNAome Atlas (NSDNA) (30) were used to explore their relation with neurodegeneation-related processes. Total read count for each miRNA by NGS is shown; For qPCR analysis, mean and 95% C.I. is shown

Key: *EVs* Extracellular vesicles, *AD* Alzheimer’s disease, *DLB* Dementia with Lewy bodies, *CSF* Cerebrospinal fluid, *PD* Parkinson’s disease, *MS* Multiple Sclerosis, *ALS* Amyotrophic lateral sclerosis, *EXs* Exosomes, *FTD* Frontotemporal dementia, *MCI* Mild cognitive impairment

### Validation phase: qPCR analysis of selected microRNAs in DLB, AD and control cohorts

**Fig. 3:**
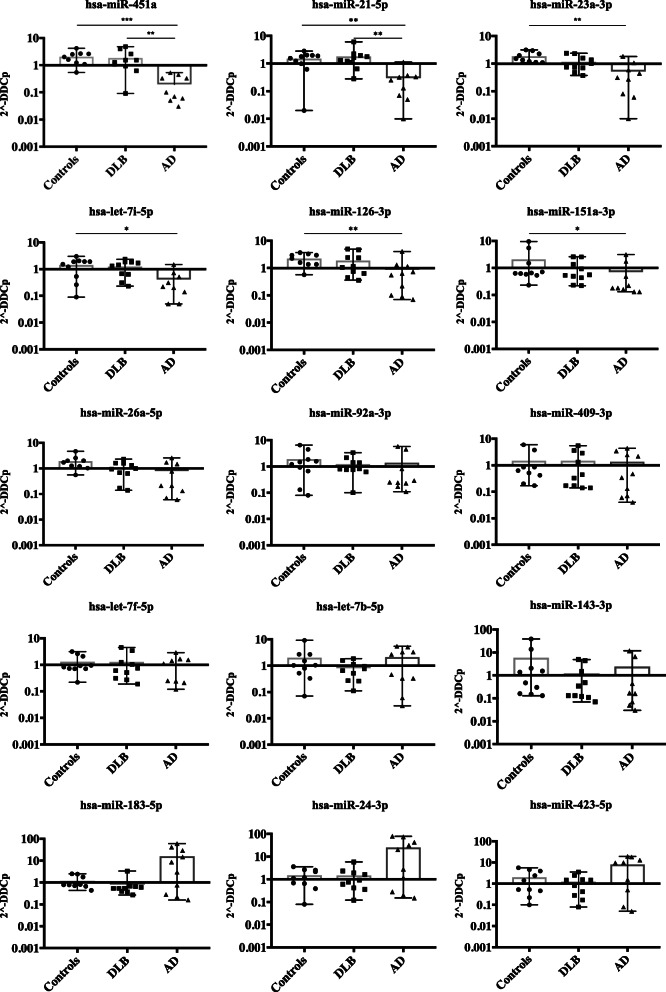
MiRNA expression levels in DLB, AD and controls. MiRNAs with highest expression differences are shown first. From the 15 analysed miRNAs, a 6-miRNA group showed significantly down-regulated expression in AD samples when compared to the two other cohorts. In all cases, mean and range for fold change are plotted; (*p < 0.05, **p < 0.005, ***p < 0.0005)

Three groups of samples including 11 DLB patients, 11 age-matched controls and 10 AD patients were used in the expression analysis by qPCR of the 15 selected miRNAs. Confirming our previous analyses by NGS, no differences were found for any of these miRNAs between DLB and healthy controls (Fig. 3).

**Fig. 4:**
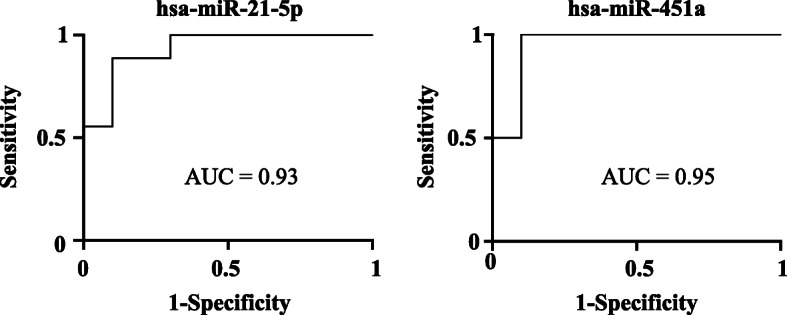
ROC curves for the differentiation between DLB and AD. Highest AUC ROC curves were obtained for hsa-miR-21-5p and hsa-miR-451a

In contrast, two miRNAs were differentially down-regulated in AD patients compared to DLB and aged-matched controls (Fig. 3) – specifically hsa-miR-451a (*p* = 0.0003 and *p* = 0.0031, vs controls and DLB respectively) and hsa-miR-21-5p (*p* = 0.0075 and *p* = 0.0064). Moreover, four miRNAs were also significantly down-regulated also in AD compared to the control cohort (Fig. 3) - hsa-miR-23a-3p (*p* = 0.0016), hsa-miR-126-3p (*p* = 0.004), hsa-let-7i-5p (*p* = 0.0154), and hsa-miR-151a-3p (*p* = 0.0335)-. The remaining nine miRNAs (out of 15) did not show any statistical difference between the three groups, though three of them showed a trend to be over-expressed in AD compared to DLB and controls –hsa-miR-183-5p, hsa-miR-24-3p and hsa-miR-423-5p (Fig. 3). A predictive diagnostic value -based on ROC curves- to discriminate between AD and DLB patients rendered high specificity and sensitivity (AUC over 0.9) for the miRNAs hsa-miR-451a and hsa-miR-21-5p (Fig. 4).

### microRNA target prediction and affected pathways

The six significantly and differentially down-regulated miRNAs in AD compared to controls and/or DLB were screened for their possible target genes using miRGate software. Up to 217 genes were predicted to be potentially regulated by these miRNAs and already validated as targets by 2 or more tools. These genes were further analysed and networked using String and Panther databases. Most of the analysed genes were related to metabolic processes (*p* = 1.6•10E-9), specifically protein metabolic processes (*p* = 5.1•10E-7). Among them, 37 were involved in the regulation of phosphorylation processes (*p* = 1.8•10E-6); specifically related to MAPK cascades and regulation of protein kinase activity (*p* = 0.009 and *p* = 1.2•10E-6, respectively). GO analysis for biological process also revealed the enrichment of response to stress (*p* = 4.3•10E-4), aging-related genes (*p* = 0.01), genes involved in neuronal morphogenesis and differentiation (*p* = 0.017 and *p* = 0.04, respectively). Particularly, several genes were related to negative regulation of neurogenesis and cell death (*p* = 0.003 and p = 0.01, respectively). Focusing in the analysis of the 2 miRNAs differentially expressed in AD vs DLB and/or controls, hsa-miR-451a and hsa-miR-21-5p, we found an enrichment of genes related to SMAD protein phosphorylation (*p* = 3.8•10E-5).

Additionally, we screened the whole list of predicted target looking for genes related to “neurodegeneration” and potentially regulated by these six miRNAs. The identified genes are listed in Table 3 and include *ADAM10* (ADAM Metallopeptidase Domain 10), *APP* (β-amyloid precursor) and *APPBP2* (amyloid protein-binding protein 2).

**Table 3:** Neurodegenerative processes-related genes among the target output from the differentially expressed miRNAs

Input Gene	Input miRNA	Start	Stop	Computational Predictions	Confirmed Predictions
*ADAM10*	hsa-let-7i-5p	783	801	Miranda	0
	hsa-miR-23a-3p	1477	1499	Miranda	0
	hsa-miR-451a	3640	3660	Miranda	0
*AKT1*	hsa-miR-451a	60	79	Miranda	Mirtarbase
*APP*	hsa-let-7i-5p	532	553	Miranda	0
*APPBP2*	hsa-let-7i-5p	3533	3553	Miranda	0
	hsa-miR-151a-3p	1868	1888	Miranda	0
	hsa-miR-21-5p	4099	4120	Miranda	0
*BCL2*	hsa-miR-126-3p	4169	4192	Miranda	0
	hsa-miR-23a-3p	4400	4418	Miranda	0
*CASP2*	hsa-let-7i-5p	2265	2285	Miranda	0
	hsa-miR-151a-3p	2025	2045	Miranda	0
*CASP3*	hsa-let-7i-5p	141	160	Miranda	0
	hsa-miR-23a-3p	808	828	Miranda	0
*CASP8*	hsa-miR-21-5p	867	888	Miranda	0
	hsa-let-7i-5p	118	139	Miranda	0
*CASP10*	hsa-miR-23a-3p	2104	2124	Miranda	0
*CASP14*	hsa-let-7i-5p	56	77	Miranda	0
*CCNE2*	hsa-miR-126-3p	1212	1233	Miranda	Mirtarbase
	hsa-miR-151a-3p	249	270	Miranda	0
	hsa-let-7i-5p	350	370	Miranda	0
*COX6B2*	hsa-let-7i-5p	69	91	Miranda	0
*COX6C*	hsa-miR-21-5p	312	332	Miranda	0
*COX7A1*	hsa-let-7i-5p	49	54	Rnahybrid	0
*COX7B*	hsa-let-7i-5p	301	321	Miranda	0
	hsa-miR-23a-3p	832	852	Miranda	0
*COX11*	hsa-miR-151a-3p	1584	1604	Miranda	0
*GSK3B*	hsa-miR-21-5p	912	934	Miranda	0
	hsa-let-7i-5p	320	342	Miranda	0
	hsa-miR-21-5p	5125	5147	Miranda	0
	hsa-miR-23a-3p	988	1008	Miranda	0
*MIF*	hsa-miR-451a	90	109	Miranda	Mirtarbase|OncomiRDB|
*UBE2J1*	hsa-miR-23a-3p	1920	1942	Miranda	0
*UBE2G2*	hsa-let-7i-5p	251	272	Miranda	0
*UBE2L3*	hsa-miR-451a	322	343	Miranda	0
	hsa-miR-23a-3p	1188	1209	Miranda	0

MirGate results for Computational and (when possible) Confirmed target genes are shown (in alphabetical order). The name of the prediction tool reporting each target gene is indicated. “0” means no confirmation was found in the analysis for that specific target. Start and Stop indicate the miRNA binding site (beginning and ending nucleotide) in the targeted gene sequence. Key: *ADAM10* ADAM Metallopeptidase Domain 10), *APP*, β-amyloid precursor), *APPBP2* Amyloid protein-binding protein 2, *GSK3B* Glycogen synthase kinase 3, *AKT1* RAC-alpha serine/ threonine-protein kinase, *CAB39* Calcium binding protein 39, *CASP* Caspase, *CCNE* Cyclin E, *COX* Cytochrome c oxidase, *MIF* Macrophage migration inhibitory factor, *UBE2* Ubiquitin Conjugating enzyme E2

## Discussion

DLB and AD show an important neuropathological, neurochemical and neuropsychiatric overlap, hampering correct DLB diagnosis, treatment and clinical management. Genetic and molecular characterization of these heterogeneous and complex disorders will lead to a better handling and diagnosis, although the definition of specific, differential and early biomarkers is still required. Plasma-EVs may become a promising reservoir of biomarkers also for neurodegenerative disorders. Besides their specific cell-derived content and the RNase-protected environment [15], EVs have been proved to cross the blood-brain barrier [10]. Therefore, although CSF would be the ideal source for specific biomarkers of central nervous system disorders, the difficulty, invasiveness, morbidity, and risk related to CSF-collection have paved the way for the analysis of plasma-derived EVs also in these pathologic scenarios.

In the current study, we analysed for the first time the miRNA content associated to plasma-EVs from DLB and AD patients compared to healthy controls. No differences in EV-markers, EV size, and morphology, or particle concentration were observed between the different cohorts. Our sequencing analysis focused on 238 miRNAs, most of them previously related to vesicles from human samples [40, 41]. Accordingly, the let-7 family accounted for around 54% of the identified miRNAs, as previously reported [77]. Overall, the NGS analyses of miRNAs did not reveal significant differences between DLB and aged-control samples.

Despite this lack of NGS significant results, a set of 15 miRNAs previously described in the literature as associated to neurodegenerative diseases and dementia were analysed by qPCR in an independent group of DLB and an additional group of AD patients compared to a different control cohort of neurologically unaffected individuals. Of notice, 10 of these miRNAs have been described among the most abundant miRNAs in the human brain [78]. No difference was observed in the expression of any of the analysed miRNAs between DLB and controls, as predicted by NGS results. However, 6 miRNAs (hsa-miR-451a, hsa-miR-21-5p, hsa-miR-23a-3p, hsa-miR-126-3p, hsa-let-7i-5p, and has-miR-151a-3p) were significantly down-regulated in AD patients compared to DLB or controls. Noteworthy, all these six miRNAs are also described as extracellular space- or exosome- associated in NCBI/Gene database. Our results are in line with previous observations on the reduced expression of hsa-miR-23a-3p in AD serum compared to controls [56] and the down-regulation of hsa-miR-126 previously described in CSF derived vesicles from AD patients [21, 51]. Besides confirming these previous studies, our expression results further indicate that DLB and AD patients may be distinguished by determining some of these miRNAs, as specifically shown by the expression profile of miRNAs hsa-miR-451a and hsa-miR-21-5p.

In comparison to healthy controls, hsa-miR-451a was previously described as down-regulated in CSF-derived AD-exosomes [55], and hsa-miR-21-5p was down-regulated in serum and CSF from AD patients [20, 42]. Therefore, both miRNAs were reported as putative biomarkers for AD. Here we confirmed the down-regulation of these two miRNAs also in plasma-derived EVs of AD patients compared to controls and, additionally to DLB. Finally, increased levels of hsa-let-7i-5p have been described in brain and CSF of AD patients [42, 50]. Also, up-regulation of hsa-miR-151a-3p has been reported in blood from AD patients in comparison to controls [44, 45]. These results are contradictory to our expression data that showed a reduced expression in AD versus controls in both cases. Having no reason to explain this later observation, it has been proposed that plasma and/or plasma-EV concentration of a given molecule including miRNA do not always show the same tendency [79], and the expression levels in peripheral circulation can also differ from those observed in CSF [42].

As a preliminary approach to evaluate the discrimination power of these miRNAs, ROC curves for the AD down-regulated miRNAs were calculated. Hsa-miR-21-5p and hsa-miR-451a rendered high AUC values to be considered a putative bio-signature for AD-DLB discrimination. Nevertheless, given the small number of samples analysed in this study, these results must be taken as tentative and as a first proof-of-concept, needing further validation in larger cohorts of patients. Likewise, it would be neither accurate to establish a specific correlation between miRNA levels and the clinical characteristics of the studied groups. Moreover, as plasma-EV associated miRNAs have been analysed, diverse causes, such as age, patient characteristics or the existence of concomitant pathologies in these aged patients, could also alter and modify these miRNAs, which seem to be deregulated.

It is known that different miRNAs can converge on the same function or be involved in the same molecular pathway. The deregulation and differential expression of these miRNAs in AD and DLB could be not only a cause or specific result of each disease, but a consequence of a common neurodegenerative process. Nevertheless, a preliminary target gene prediction for the 6 down-regulated miRNAs in our study defined a group of 217 most confirmed target genes (with more than 2 confirmed predictions), mostly implicated in metabolic processes and protein phosphorylation. The role of protein-phosphorylation during neurodegeneration has been widely described as important in the spread and accumulation of α-synuclein in DLB or PD brains [80, 81]. Also, the increased levels of phosphorylated tau in AD have been considered as an important marker of AD pathogenesis [82]. Specifically, genes involved in the Pl3K-AKt pathway, such as *CAB39* [83], are among the predicted genes in our analysis. Together with *AKT1*, *CAB39* would be possibly regulated by hsa-miR-451a. Therefore, our data suggest an impairment of this pathway, involved in neuron differentiation-proliferation and death [84]. Other genes related to neurodegeneration were also found, although with a lower prediction rate (Table 3). For instance, *ADAM10, APPBP2* and *APP,* identified as hsa-let-7i-5p targets, are directly involved in the intracellular transport and deposition of β-amyloid peptides [85]. As inferred from our results, the reduction of hsa-miR-let-7i-5p, together with hsa-miR-21-5p and hsa-miR-151a-3p, could alter *APPBP2* and *APP* expression resulting in an incorrect APP cleavage and promoting β-amyloid accumulation. Other genes like *GSK3B* (Glycogen synthase kinase 3) may play a role in neuroinflammation [86], neuronal apoptosis and accumulation of phosphorylated tau - in AD [87]. In our study, several miRNAs targeting *GSK3B* were down-regulated in AD samples. Furthermore, cell-death related genes, such as *BCL2,* cyclins involved in cell cycle or genes involved in the degradation of selective proteins (including β-amyloid peptide) such as proteasomal proteins were also present in the predicted network. The impairment of the proteasomal pathway would increase β-amyloid deposition and promote AD pathology [88]. Of notice, among the miRNAs and possible target genes differentially regulated in DLB vs AD (hsa-miR-21-5p and hsa-miR-451a) we found genes related to SMAD protein phosphorylation. The role of SMAD proteins in AD has been described, by the presence of smad2 within amyloid plaques and neurofibrillary tangles [89].

Altogether our data point to a specific reduced expression of AD-related miRNAs that would target genes primarily involved in protein phosphorylation cascades and the neuropathology of AD. The differential expression of these miRNAs in AD versus DLB patients could be considered as a putative biomarker for the identification/discrimination between both disorders, which could help neurologist to overcome the clinical and pathological overlap between AD and DLB. Nevertheless, as a first exploratory study, these data have to be further confirmed in larger cohorts of patients.

## Conclusions

To our knowledge, this is the first study on the comparison of the miRNA profile associated to plasma-EVs from DLB and AD patients. Despite the limited number of samples, our study provides preliminary evidence for different miRNA expression levels between the two most common types of degenerative dementia, with changes related to target genes and pathways involved in the pathogenesis of AD.

## Additional files

Additional file 1: Purchasing information about miRCURY LNA™ Universal RT microRNA PCR, Pick-&-Mix, Ready-to-use Panels used in the study. (XLSX 53 kb)

Additional file 2: Raw data from the Next Generation Sequencing experiment for DLB and control samples. (XLSX 84 kb)

## Data Availability

The datasets used and/or analysed during the current study are available from the corresponding author on reasonable request.

## Abbreviations

AD: Alzheimer’s disease
ADAM10: ADAM Metallopeptidase Domain 10)
AKT1: RAC-alpha serine/ threonine-protein kinase
ALS: Amyotrophic lateral sclerosis
APP: β-amyloid precursor
APPBP2: Amyloid protein-binding protein 2
AUC: Area under the curve
CAB39: Calcium binding protein 39
CASP: Caspase
CCNE: Cyclin E
COX: Cytochrome c oxidase
Cq: Quantification cycle
Cryo-EM: Cryo-electron microscopy
CSF: Cerebrospinal fluid
DLB: Dementia with Lewy bodies
EVs: Extracellular vesicles
EXs: Exosomes
FTD: Frontotemporal dementia
GSK3B: Glycogen synthase kinase 3
ISEV: International Society for Extracellular Vesicles
KEEG: Kyoto Encyclopedia of Genes and Genomes
LOO: Leave-one-out
MCI: Mild cognitive impairment
MFI: Mean fluorescence intensity
MIF: Macrophage migration inhibitory factor
miRNA: Micro RNA
MS: Multiple sclerosis
MVs: Microvesicles
NGS: Next Generation Sequencing
NSDNA: The Nervous System Disease NcRNAome Atlas
PD: Parkinson’s disease
ROC: Receiver Operating Characteristic
SD: Standard desviation
SEC: Size-Exclusion chromatography
TMM: Trimmed means of M-values
UBE2: Ubiquitin Conjugating enzyme E2
